# *APOE4* genotype shapes the role of dietary fibers in cognitive health through gut microbiota changes

**DOI:** 10.1080/19490976.2025.2526133

**Published:** 2025-07-02

**Authors:** Marrium Liaquat, Gwenaelle Le Gall, Andrew Scholey, Matthew G. Pontifex, Thomaz F.S. Bastiaanssen, Michael Muller, Anne Marie Minihane, David Vauzour

**Affiliations:** a Norwich Medical School, University of East Anglia, Norwich, UK; b Centre for Human Psychopharmacology, Swinburne University, Hawthorn, Australia; c Department of Psychology, Northumbria University, Newcastle upon Tyne, UK; d Amsterdam University Medical Center, University of Amsterdam, Amsterdam, Netherland

**Keywords:** *APOE* genotype, cognitive status, short-chain fatty acids, microbiome, SCI, MCI

## Abstract

*APOE4*, a key risk factor for Alzheimer’s disease, influences gut microbiota and microbial metabolites (e.g. amino acids and dietary fiber (DF) derived short-chain fatty acids (SCFAs)). However, its role in modulating microbiota-driven DF metabolism and its effect on cognitive status remains unclear. This cross-sectional study (*n* = 170) investigates the association between *APOE4* genotype, DF consumption, and metabolism in individuals with subjective cognitive impairment (SCI) and mild cognitive impairment (MCI) compared to healthy controls (HC). Liquid Chromatography Tandem Mass Spectrometry (LC-MS/MS) and ^*1*^H NMR metabolomic techniques were used to quantify SCFAs in serum and fecal samples, respectively. Gut microbiota speciation was carried out by 16S rRNA amplicon sequencing. We found that DF intake was significantly associated with *APOE4* genotype and cognitive status, with lower consumption in *APOE4* carriers (*p* < 0.05) and those with cognitive impairment (SCI and MCI) (*p* = 0.03). Differences (*p* < 0.05) in gut microbiota (both α- and β-diversity) and SCFAs were evident between *APOE4* and non-*APOE4* carriers, with stronger associations with DF consumption and cognitive status evident in non-*APOE4* carriers. These findings suggest that targeting DF-induced changes in gut microbiota and serum SCFAs may be an effective strategy for mitigating cognitive impairment, but primarily in non-*APOE4* carriers.

## 1. Introduction

The *APOE4* genotype is the strongest genetic risk factor for Alzheimer’s disease (AD) and also for earlier stages like mild Cognitive Impairment (MCI).^1^ Evidence suggests that *APOE4* genotype influences cognition even in pre-MCI state such as subjective cognitive impairment (SCI).^2^ Systemic and central metabolic dysfunction associated with this neuropathology is evident often decades before any symptoms appear.^3^ *APOE4* carriers also present with a distinct gut-microbial diversity which potentially contributes to the pathological metabolic effects of *APOE4* genotype.^3–7^

The intestinal microbiome^8–10^ plays a key role in various physiological changes that contribute to cognitive decline via disrupted gastrointestinal function, weakened gut barrier integrity, and alterations in intestinal neurotransmitters and immune markers,^11^ which bi-directionally communicate with the brain.^12,13^ Therefore, the gut microbiota is emerging as a tractable target to affect cognitive functions thorough microbiome-gut-brain axis mainly via microbial metabolites.^14,15^

Microbiome research has unveiled the role of dietary fibers (DFs) in modulating the host microbiota and associated metabolites such as the short-chain fatty acids (SCFAs) in human health and disease.^16^ Different types of DFs offer various metabolic benefits to the host, largely determined by their physicochemical properties, such as solubility, viscosity, and fermentability, which also serve as the basis for many DF classification systems.^16,17^ A key aspect of this relationship is understanding how each specific type of DF shapes the composition of the host’s microbiota.^16^ Neuroprotective diets like the Mediterranean diet (MedDiet), Dietary Approaches to Stop Hypertension (DASH), and the Mediterranean-DASH diet Intervention for Neurodegenerative Delay (MIND diet) have in common a high consumption of DF and fiber-rich foods like whole grain cereals, fruits, vegetables, legumes, and nuts. These dietary patterns modify various biological processes involved in cognitive decline including neuroinflammation, oxidative stress, hippocampal neurogenesis impairment, neurovascular dysfunction, and gut microbiota dysbiosis.^18^

The biological mediators driving these physiological effects remain largely unknown; SCFAs are the microbial metabolites from bacterial fermentation of DF in the intestine and are often considered key candidate mediators. SCFAs might be directly or indirectly involved in communication along the Microbiome-Gut-Brain axis because of their direct neuroactive properties or involvement in gut – brain signaling pathways through immune and endocrine systems.^12^ One such SCFA, butyrate, has been reported to influence the release of serotonin and gut hormones in the enteric nervous system which can stimulate the vagus nerve and trigger endocrine signaling impacting brain function.^19^ SCFAs also exert several effects locally to improve gut health such as the maintenance of intestinal barrier integrity and protection from intestinal inflammation. SCFAs can also cross the blood–brain barrier (BBB) via monocarboxylate transporters (MCTs) abundantly expressed on endothelial cells^12^ and potentially centrally active. Reduced abundance of SCFA-producing bacteria is associated with higher odds of AD pathology,^20^ with similar gut microbiota changes also reported in cognitive impairment.^9,21,22^

There is substantial evidence that *APOE4* genotype and cognitive ‘health’ are associated with the gut microbiome composition and metabolism, and DF is a promising substrate for modulating gut-bacterial diversity and ultimately cognitive function. Therefore, gut microbiota targeted interventions, such as increasing DF intake, may be a promising approach to promote beneficial changes in the gut microbiome. However, currently, the role of DF and DF-derived microbial metabolites (SCFAs) and their associations with cognitive status and *APOE4* genotype are unknown and the subject of the current analyses.

Therefore, in this study, we investigate the associations between *APOE4* genotype status, DF intake, and SCFA profiles in those with SCI or MCI, compared to healthy controls (HC).

## 2. Materials and methods

### 2.1. Subjects and methods

The study participants were recruited for the CANN (Cognitive Ageing, Nutrition and Neurogenesis) and COMBAT (The impact of Cranberries On the Microbiome and Brain in healthy Ageing sTudy) trials registered in clinicaltrials.gov as NCT02525198 and NCT03679533, respectively. The CANN study recruited 259 participants, aged ≥50 years, with SCI or MCI based on criteria developed by the National Institute of Aging-Alzheimer’s Association, with no indication of clinical dementia.^23^ Briefly, the classification of MCI was based on established criteria, requiring (1) self-reported memory decline over 2–3 years, (2) preserved functional independence, indicated by a Functional Activities Questionnaire score of less than 6, (3) absence of dementia, defined as a Montreal Cognitive Assessment score of 18 or higher,^24^ and (4) no significant depression, determined by a Geriatric Depression Scale-15 score of less than 10.^25^ Additionally, MCI classification required cognitive impairment of at least one standard deviation below the age- and education-adjusted mean on at least one neuropsychological test assessing memory (California Verbal Learning Test-II^26^), language (Boston Naming Test^27^), visuospatial function (Figure Copy task of the Repeatable Battery for the Assessment of Neuropsychological Status^28^), attention (Digit Span task, Forward or Backward, from the Wechsler Adult Intelligence Scale^29^), or executive function (Trail Making Test, Part A or B^30^). Participants meeting all criteria except for cognitive impairment were classified as SCI. Cognitively healthy adults were selected from the COMBAT study as a control group. The COMBAT study recruited 60 adults, aged 50–80 years, with no subjective memory complaints as assessed by the Cognitive Change Index (CCI) questionnaire.^31^ All groups (HC, SCI, MCI) were matched for age, sex, and BMI. The participants were grouped based on *APOE* genotype, into *APOE4* carriers (E3/E4 and E4/E4, with E2/E4 excluded) and non-*APOE4* carriers (E3/E3 and E2/E3). The participants were split (independent of their cognitive status) into tertiles based on their DF consumption, into low (<15.03 g/day), medium (15.03–20.85 g/day), and high (20.85–41.35 g/day) consumers. Fecal samples were collected during the baseline visit using the collection vessels (NHS-approved Easy sampler collection kit). The storage container was placed in a cool dry location prior to returning to the research facility at the earliest opportunity (i.e. at the study visit) and subsequently stored at −80°C until analysis.

### 2.2. Chemicals and reagents

All SCFA standards, namely acetic acid (C2:0), propionic acid (C3:0), butyric acid (C4:0), isobutyric acid (C4:0), 2-methylbutyric acid (C5:0), valeric acid (C5:0), isovaleric acid (C5:0), caproic acid (C6:0), and isocaproic acid (C6:0), were purchased from Merck (UK). Acetic acid d-4, propionic acid d-2, and 2-isobutoxyacetic acid were used as internal standards. For derivatization, 3-nitrophenylhydrazine hydrochloride (3-NPH), *N*-(3-dimethylaminopropyl)-N’-ethylcarbodiimide hydrochloride (EDC), and pyridine were purchased from Merck (UK). All the solvents used in the experimental procedures were of HPLC grade.

### 2.3. LCMS\MS-based quantification of serum SCFAs

Sera SCFAs were quantified by targeted metabolomics using Liquid Chromatography Tandem Mass Spectrometry (LC-MS/MS) combined with Multiple Reaction Monitoring (MRM) mode. Extraction and derivatization of SCFAs was adapted from literature and further modified.^32^ 40 *µ*L serum was diluted with 500 *µ*L ice-cold methanol and incubated on dry ice for 15 min. The samples were centrifuged (14800 rpm, 5 min) and supernatants were filtered using 0.45 *µm* PTFE filters. The filtered extracts were evaporated until dryness using a Savant™ SpeedVac™ High-Capacity Concentrator (Cat. SC210A–230) and reconstituted with 40 *µ*L of methanol. 20 *µ*L of reconstituted sample was mixed with 20 *µ*L of internal standard mix (20ppm ac-d4, pro-d2, isobutoxyacetic acid). For derivatization, 10 *µ*L of 3-NPH (~50 mM 3-NPH solution) and 10 *µ*L of EDC (~50 mM EDC solution) made in 7% pyridine (v/v methanol) were added to the reconstituted extracts and incubated at 37°C for 30 min in a standard incubator (Model B 28, Binder, Tuttlingen, Germany). The derivatization reaction was quenched by adding 20 *µ*L of 0.1% formic acid before being run on the LC-MS/MS system.

The analytical system consisted of Waters Acquity UPLC equipped with Water Xevo TQ-S mass spectrometer that is controlled by MassLynx v4.2 software for data acquisition, analyses, and data management. Chromatographic separation was achieved on Kinetex® 2.6 *µm* XB-C18, 50 × 2.1 mm column (Phenomenex, Torrance, CA, USA). Column temperature was maintained at 60°C with 0.1% formic acid in water as mobile phase A and 0.1% formic acid in acetonitrile as mobile phase B, with a constant flow rate of 0.5 mL/min. The elution gradient began at 90% A, which linearly decreased to 50% at 6 min. From 7 to 7.5 min, solvent B was 100%, which was maintained at 10% hereafter until 13 min.

### 2.4. ^*1*^H NMR fecal metabolomic analysis

Fecal metabolites were analyzed and quantified using ^*1*^H NMR metabolomics analysis. The preparation method was similar to that reported previously.^6^ Briefly, 50 mg of human fecal sample was mixed with 800 *µl* of NMR buffer [0.26 g Na_2_HPO_4_, 1.44 g K_2_HPO_4_, 17 mg TSP (sodium 3-(trimethylsilyl)-propionate-d4 (3-Trimethylsilyl) propionic-2,2,3,3-d4 acid sodium salt) in 100 ml deuterated water]. The samples were vortexed until completely homogenized. After mixing and centrifugation (15,000 g for 5 min), 550 *µl* of the supernatant was transferred to a 5-mm NMR tube for spectral acquisition. NMR spectra were recorded on a 600-MHz Bruker Avance spectrometer fitted with a 5-mm TCI proton-optimized triple resonance NMR inverse cryoprobe and a 24-slot autosampler (Bruker, Coventry, England). Sample temperature was maintained at 300 K (27°C). Each spectrum consisted of 128 scans of 65,536 complex data points with a spectral width of 20 ppm (acquisition time 2.6 s).

The noesypr1d presaturation sequence was used to supress the residual water signal with low power selective irradiation at the water frequency during the recycle delay (D1 = 4s) and mixing time (D8 = 0.01s). A 90° pulse length of 8.1 *µ*s was set for all samples. Spectra were transformed with a 0.3-Hz line broadening and zero filling, manually phased, baseline corrected, and referenced by setting the TSP methyl signal to 0 ppm. Metabolite identification and quantification was done using the software Chenomx (V 8.6).

### 2.5. Microbial DNA extraction and 16S rRNA amplicon sequencing

Microbial DNA was extracted from 150 to 250 mg fecal samples using QIAamp PowerFaecal Pro DNA kit (Qiagen) following the manufacturer’s instructions. Extracted DNA samples were quantified using NanoDrop (ND-1000 spectrophotometer). Agarose gel electrophoresis was used for quality assessment, which distinguished DNA integrity, purity, fragment size, and concentration. The V3-V4 hypervariable region of the 16S rRNA gene was amplified with Illumina NovaSeq 6000 PE250 platform. Sequence analysis was performed using the Uparse software (Uparse v7.0.1001), using all the effective tags. Sequences with ≥97% similarity were assigned to the same Operational Taxonomic Unit (OTU). A representative sequence for each OTU was screened for further annotation. Each representative sequence was aligned against the SSuRNA database of SILVA database 138.^33^ Alpha diversity was assessed using Chao1, Shannon, and Simpson diversity indices, while beta diversity was assessed using Aitchison distance.

### 2.6. Dietary assessment

Baseline dietary assessment was conducted using the validated European Prospective Investigation into Cancer and Nutrition FFQ^23^ and Scottish Collaborative Group FFQ version 6.6^34^ for the CANN and COMBAT cohorts, respectively. Nutrient and fiber intake was established from the FFQ data using the seventh edition of McCance and Widdowson food composition tables.^35^

### 2.7. Statistical analyses

Statistical analyses were performed using RStudio version 4.4.0 and GraphPad Prism version 10 (GraphPad Software, CA, USA). All data are presented as mean ± standard deviation (SD) unless otherwise mentioned. After identifying outliers (ROUT method, q = 1%) metabolomics data from both serum LCMS/MS and fecal NMR analyses were log transformed and analyzed using Mann–Whitney tests or two-way ANOVA, followed by Benjamini and Hochberg method for FDR correction. Spearman correlations were performed to assess associations among metabolites, dietary intake, and bacterial abundance.

Microbiome analyses were performed on CLR (centered log-ratio) transformed data after filtering the datasets excluding all genera and features with <10% prevalence, except for alpha diversity metrics where we are interested in rare taxa. Alpha diversity was measured after adjusting for rarefaction curves, and a linear model approach was used to compare differences in alpha diversity metrics within a group, while accounting for the effect of other variables.

Beta diversity was reported after pairwise Permutational Multivariate Analysis of Variance (PERMANOVA) testing on Aitchison distance values (Euclidean distance over CLR-transformed values) to assess microbiota divergence between groups, allowing 1,000 permutations. Ordinations were built using Principal Component Analysis on Aitchison distance values, and the PCA plots present variance (R^2^) and *p* values for each plot (Figure 4). Gut-Brain Modules (GBMs) and Gut-Metabolic Modules (GMMs) were identified based on PICRUSt2 predictions, which are based on several gene family databases including Kyoto Encyclopedia of Genes and Genomes (KEGG) orthologs (KO), Enzyme Commission (EC) numbers, and MetaCyc pathway abundances.^36^ These PICRUSt2 outputs have been validated with various human microbiome datasets.^37^ GBM modules are annotated for function, pathway, structure, and potential to cross the intestinal epithelium and the blood–brain barrier, and their abundances were derived from an orthologue abundance table using Omixer Reference Pathways Mapper.^38^ The same was done for GMMs.^39^ GBM and GMM counts were CLR-transformed after removing the features with <10% prevalence, followed by fitting a linear regression model and FDR correction using Benjamini and Hochberg method.

Differential bacterial abundance analyses were performed using linear models built for each genus to test multiple transformations accounting for different data distribution. *p* values for all the contrasts were corrected for False Discovery Rate (FDR) using Benjamini & Hochberg’s procedure. q-values of 0.1 and 0.2 were used as a cutoff for statistical significance for genera and functional modules, respectively. CLR transformed data was reported for the final figures as presented.

## 3. Results

### 3.1. Participant characteristics

A total of 170 participants were included in the analyses, comparing 73 SCI and 60 MCI participants with 37 older healthy adults as controls (Table 1). The mean ± SD age and BMI of the participants was 65.8 ± 6y and 25.7 ± 3.7 kg/m^2^. Overall, 56% were female and 44% were *APOE4* carriers. The participants in each DF tertile group were matched for age, sex, and BMI between *APOE4* and non-*APOE4* carriers, except Low DF consumer group (where they were not matched for sex, due to limited number of participants) (Supp Table S2).

**Table 1.** Baseline characteristics of the participants.

	Overall	Low DF Consumers (*n* = 57)		Medium DF Consumers (*n* = 56)		High DF Consumers (*n* = 57)	
		Non-*APOE4* carriers	*APOE4* carriers	Non-*APOE4* carriers	*APOE4* carriers	Non-*APOE4* carriers	*APOE4* carriers
n		29	28	28	28	39	18
HC	37	10	3	4	2	17	1
SCI	73	7	14	14	19	14	5
MCI	60	12	11	10	7	8	12
Age (y)	65.8 (6.0)	65.2 (6.7)	64.8 (6.3)	66.4 (4.4)	66.0 (6.4)	65.8 (6.1)	66.6 (6.0)
Sex, M/F (%F)	75/95 (56%)	7/22 (76%)	12/16 (57%)	15/13 (46%)	11/17 (61%)	20/19 (49%)	10/8 (44%)
BMI (kg/m^2^)	25.7 (3.7)	24.9 (3.0)	28.0 (4.5)	25.1 (3.0)	25.9 (4.6)	25.4 (2.8)	25.2 (3.7)

Data are mean (SD) or as stated otherwise.

DF, dietary fiber; HC, healthy controls; SCI, subjective cognitive impairment; MCI, mild cognitive impairment.

### 3.2. *APOE* genotype and cognitive status influence dietary fiber intake and circulating SCFAs

**Figure 1.**
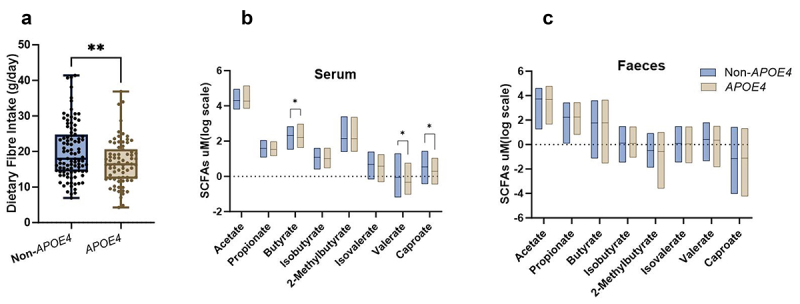
Association between *APOE4* genotype, dietary fiber (DF) intake and SCFAs concentrations. a) DF intake (g/day) in non-*APOE4* and *APOE4* carriers. b) Serum and c) fecal SCFAs concentrations (*µ*M log scaled) in individuals with non-*APOE4* and *APOE4* genotype. Statistical comparisons were done by Mann–Whitney U tests and multiple comparisons were adjusted for false discovery rate (FDR). Data are represented as mean ± standard deviation. * = p < 0.05 and ** = p < 0.01.

*APOE4* carriers consumed on average 16% less DF compared to non-*APOE4* carriers (F (1, 73) = 7.295; *p* = 0.009, Figure 1(a)), which corresponded with lower circulating SCFAs (Figure 1(b)). Specifically, serum levels of butyrate, valerate, and caproate were significantly lower in *APOE4* carriers (Figure 1(b)). No differences were observed in fecal SCFAs concentrations between the genotypes (Figure 1(c)). DF consumption did not affect either serum (Supp Fig S1a) or fecal SCFAs levels (Supp Figure 1(b)) which confirmed that the observed variation in serum SCFAs between *APOE4* and non-*APOE4* carriers is independent of DF intake. Additionally, we were also interested to see whether the response to DF intake is *APOE4* genotype dependent. No significant associations between DF intake and either serum (Supp Fig S1c) or fecal (Supp Fig S1d) level of SCFAs were evident in either non-*APOE4* or *APOE4* carriers.

**Figure 2.**
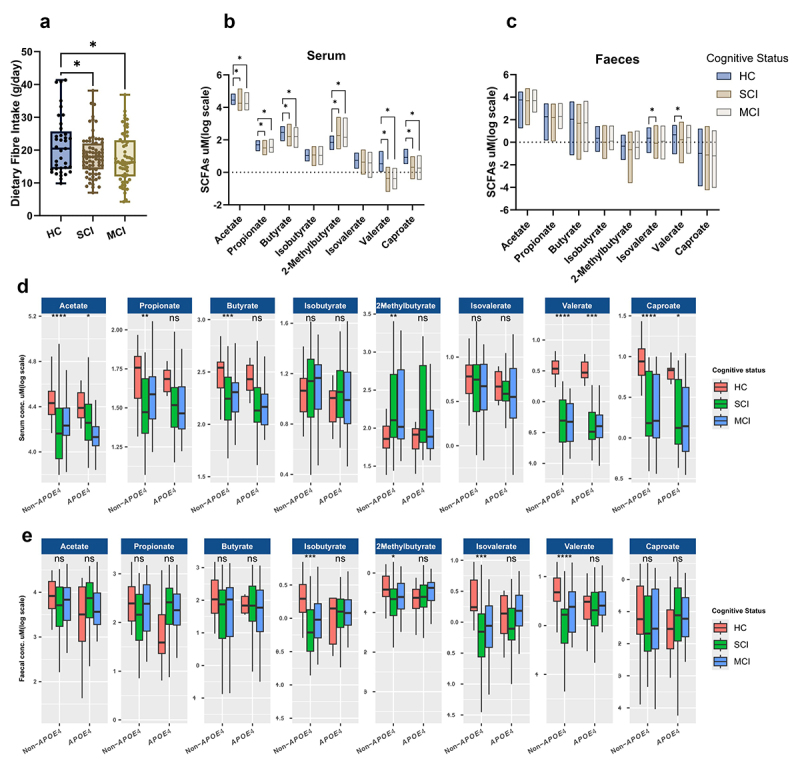
Association between cognitive status, *APOE4* genotype, dietary fiber (DF) intake and SCFAs concentrations. (a) DF intake (g/day) in healthy controls (HC), subjective cognitive impairment (SCI) and mild cognitive impairment (MCI) participants. (b) Serum and (c) fecal SCFAs concentrations (*µ*M log scaled) in HC, SCI, and MCI participants. d) serum SCFAs concentrations (*µ*M log scaled) in individuals with non-*APOE4* and *APOE4* genotype and split based on their cognitive status. e) Fecal SCFAs concentrations *(µ*M log scaled) in individuals with non-*APOE4* and *APOE4* genotype and split based on their cognitive status. Statistical comparisons were done by Kruskal–Wallis test and significance levels are presented on the plots; ns = non-significant (*p* > 0.05), * = p < 0.05, ** = p < 0.01, ***= *p* < 0.001.

The cognitive status of individuals was also associated with DF consumption. Compared to SCI and MCI participants, healthy controls consumed 18% and 22% more DF, respectively (F (2, 167) = 3.568; *p* = 0.03; Figure 2(a)). This was also mirrored by significantly lower serum levels of straight SCFAs (*p* < 0.05) in cognitively impaired individuals, including acetate, propionate, butyrate, valerate, and caproate (Figure 2(b)). Contrarily, a branched form of butyrate, 2-Methylbutyrate was significantly higher (*p* < 0.05) in cognitively impaired individuals as compared to healthy controls (Figure 2(b)). Fecal levels of SCFAs were only significantly different for C-5 isovalerate and valerate (*p* < 0.05) between healthy and SCI individuals (Figure 2(c)). These differences in SCFAs were also independent of DF intake as confirmed for *APOE4* genotype.

To establish if *APOE4* genotype influences how cognitive status relates to SCFA levels, we split the cohort based on their *APOE4* genotype and observed that, non-*APOE4* carriers had more significant associations between serum SCFAs and cognitive status, as compared to *APOE4* carriers (Figure 2(d)). Cognitive status was also associated with fecal SCFAs differences in non-*APOE4* carriers only, for isobutyrate, 2-methylbutyrate, isovalerate, and valerate (Figure 2(e)).

### 3.3. Fecal SCFAs do not reflect circulating SCFA levels and dietary fibre intake

**Figure 3.**
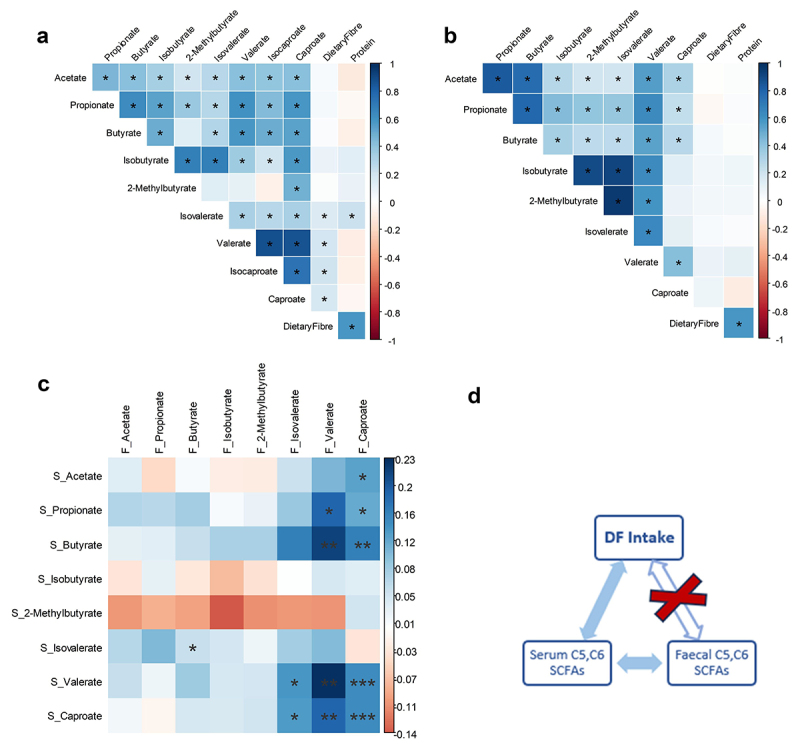
Serum and fecal SCFAs correlation with dietary fiber (DF) and protein intake. (a) Serum SCFAs correlation with DF and protein intake; (b) fecal SCFAs correlation with DF and protein intake. c) Correlation between serum and fecal SCFAs. Spearman correlations are plotted, and significant correlations are annotated as *=p < 0.05, **=p < 0.01, and ***=p < 0.001. (d) Association between DF intake and C5, C6 SCFAs, blue arrows indicate significant positive association and red cross (x) indicates non-significant association.

No significant difference in either serum or fecal levels of SCFAs based on DF intake was observed (Supp Fig S1a,b). DF intake was not correlated (*p* > 0.05) with either serum (Figure 3(a)) or fecal (Figure 3(b)) SCFAs; except with serum isovaleric (R^2^ = 0.16, *p* < 0.05), valeric (R^2^ = 0.19, *p* < 0.05), isocaproic (R^2^ = 0.20, *p* < 0.05), and caproic (R^2^ = 0.17, *p* < 0.05) acids. Protein as the primary precursor of branched SCFAs was positively correlated with serum isovaleric levels (R^2^ = 0.21, *p* < 0.05, Figure 3(a)) only. No correlations were observed between protein or DF intake and fecal SCFAs.

Fecal C5-C6 SCFAs were correlated (*p* < 0.05) with serum C5-C6 SCFAs which is supported by their minimal utilization in the gut and liver. However, these fecal C5-C6 SCFAs are also positively correlated with serum C2-C4 (acetate, propionate, and butyrate) SCFAs (Figure 3(c)), which is possibly due to metabolism of serum C5-C6 SCFAs to serum C2-C4 SCFAs, which represents an indirect association between serum C5, C6 SCFAs, and DF intake (Figure 3(d)).

### 3.4. Differential influence of *APOE4* genotype and DF intake on gut microbiota and circulating SCFAs

**Figure 4.**
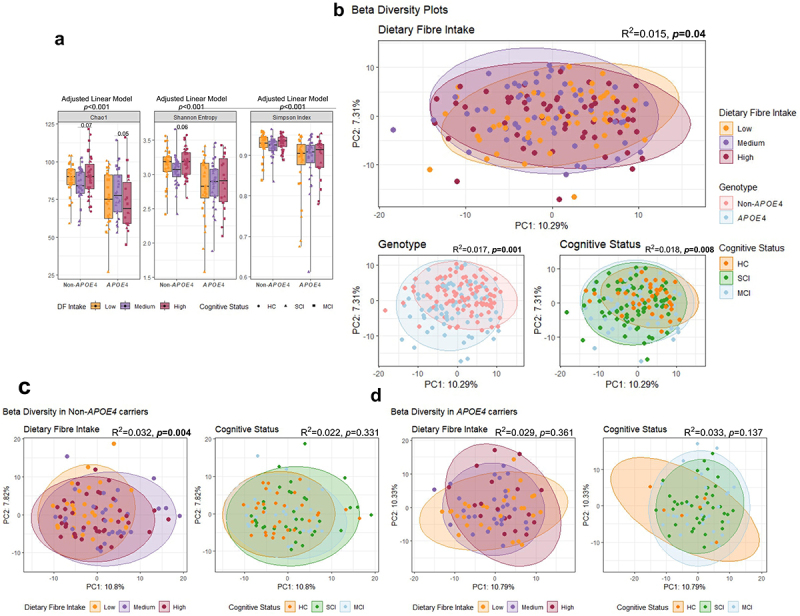
Alpha and beta diversity indices and associations between *APOE4* genotype, cognitive status, and dietary fiber (DF) intake. (a) Alpha diversity indices including Chao1, Shannon Entropy and Simpson index, (b) PCA plots presenting β-diversity changes based on dietary fiber (DF) intake, genotype, and cognitive status, (c) PCA plots presenting β-diversity in non-*APOE4* carriers based on DF intake and cognitive status. (d) PCA plots presenting β-diversity in *APOE4* carriers based on DF intake and cognitive status. Linear regression model was used for alpha diversity analyses between genotypes while adjusting for participants’ cognitive status and DF intake; *p* values are plotted on top of the plots. Alpha diversity comparisons within each genotype were adjusted for participants’ cognitive status and select *p* values reported on the plots. PERMANOVA analyses were performed on Aitchison distance values for β-diversity; variance (R^2^) and *p* values are plotted on each PCA plot.

*APOE4* carriers had significantly lower alpha diversity, as measured by Chao1 (*p* < 0.001), Shannon Entropy (*p* < 0.001) and Simpson index (*p* < 0.001), compared to non-*APOE4* carriers (Figure 4(a)). PERMANOVA analysis demonstrated that beta diversity was also significantly influenced by *APOE4* genotype (R^2^ = 0.017; *p* = 0.001, Figure 4(b)).

Non-*APOE4* carriers responded differently to DF intake as reflected by their α and β-diversity indices. Although DF intake influenced species richness (measured by Chao1 index) in both non-*APOE4* (*p* = 0.07) and *APOE4* (*p* = 0.05) genotypes, species evenness (measured by Shannon Entropy) was only influenced in non-*APOE4* carriers (albeit not significantly, *p* = 0.06) (Figure 4(a)). Participants with medium DF consumption exhibited higher α-diversity (Chao1 and Shannon Entropy indices) among *APOE4* carriers and lower α-diversity among non-*APOE4* carriers compared to low or high DF consumers (Figure 4(a)).

β-diversity was also significantly influenced by DF intake (R^2^ = 0.015; *p* = 0.04; Figure 4(b)) and pairwise PERMANOVA identified a significant difference between medium and high DF consumers (R^2^ = 0.013; *p* = 0.04) only. However, splitting the cohort based on their *APOE* genotype showed that the influence of DF intake on β-diversity was solely within the non-*APOE4* carrier subgroup (R^2^ = 0.032; *p* = 0.004; Figure 4(c)) versus *APOE4* carriers (R^2^ = 0.029; *p* = 0.361; Figure 4(d)). Non-*APOE4* carriers had a significant difference between low vs medium DF consumers (R^2^ = 0.03; *p* = 0.005) and medium vs high DF consumers (R^2^ = 0.03; *p* = 0.02).

β-diversity was also significantly influenced by cognitive status (R^2^ = 0.018; *p* = 0.008) (Figure 4(b)). PERMANOVA analysis highlighted that HC showed significant difference in β-diversity compared to cognitively impaired groups; HC vs SCI (R^2^ = 0.02; *p* = 0.003) and HC vs MCI (R^2^ = 0.02; *p* = 0.03). However, there was no difference between SCI and MCI. *APOE4* genotype had no influence on the association of cognitive status in changing β-diversity (Fig c, d).

**Figure 5.**
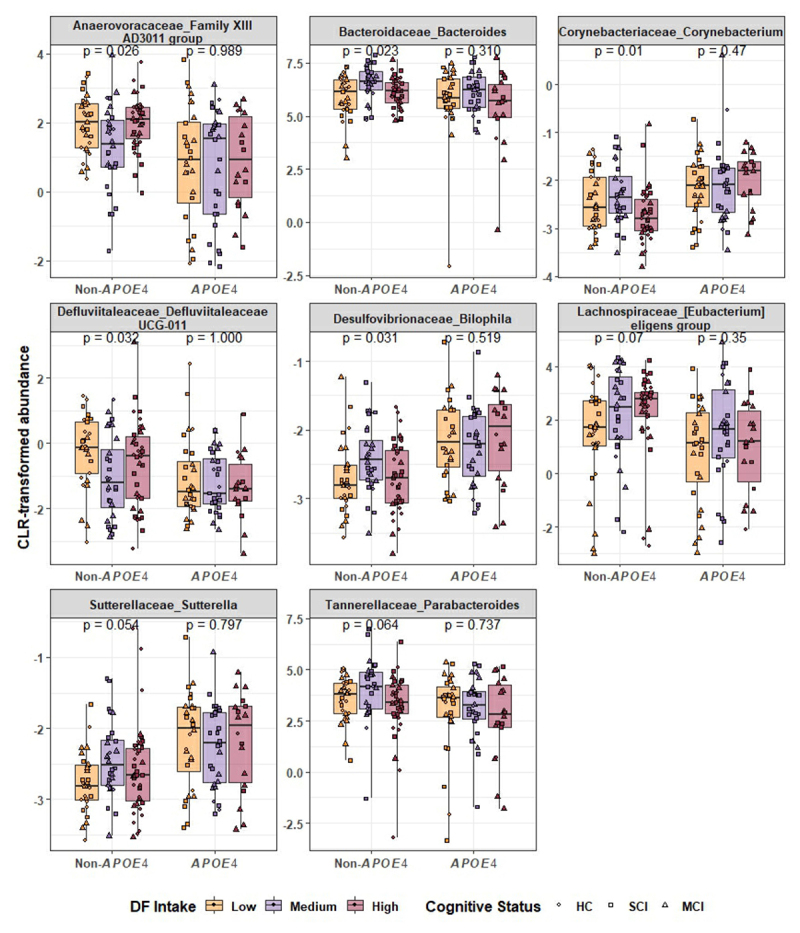
Association between dietary fiber (DF) intake and abundance of bacterial genera within non-*APOE4* and *APOE4* carriers. Kruskal–Wallis test was performed to compare the effect of DF intake within each genotype, *p* values are reported within individual boxplots.

Various bacterial genera were differentially abundant between non-*APOE4* and *APOE4* carriers (Supp Table S1), which is consistent with the significant β-diversity differences between both genotypes. This differential bacterial abundance was affected by DF intake (Supp Fig S2), with significant changes observed within non-*APOE4* carriers only (Figure 5). *Bacteroides* and *Parabacteroides* were higher in non-*APOE4* carriers (FDR = 0.04 and 0.03, respectively, Supp Table S1). *Bacteroides* genera is one of the most common DF degraders in the gut and was significantly (*p* = 0.02) higher in higher DF tertiles in non-*APOE4* carriers only, although this increase was not linear (Figure 5). A comparable opposite trend was observed in *Parabacteroides* abundance (*p* = 0.06) in non-*APOE4* carriers only (Figure 5). In contrast, higher DF intakes were associated with a lower abundance of *Corynebaterium* (*p* = 0.01) and *Defluviitaleaceae UCG-011* (*p* = 0.03) in non-*APOE4* carriers, whereas no significant difference was observed in *APOE4* carriers. *Sutterella* and *Bilophila* levels were also lower in non-*APOE4* compared to those with the *APOE4* allele. In non-*APOE4* carriers, both these genera showed significantly higher but non-linear abundance with higher DF intake (Figure 5).

**Figure 6.**
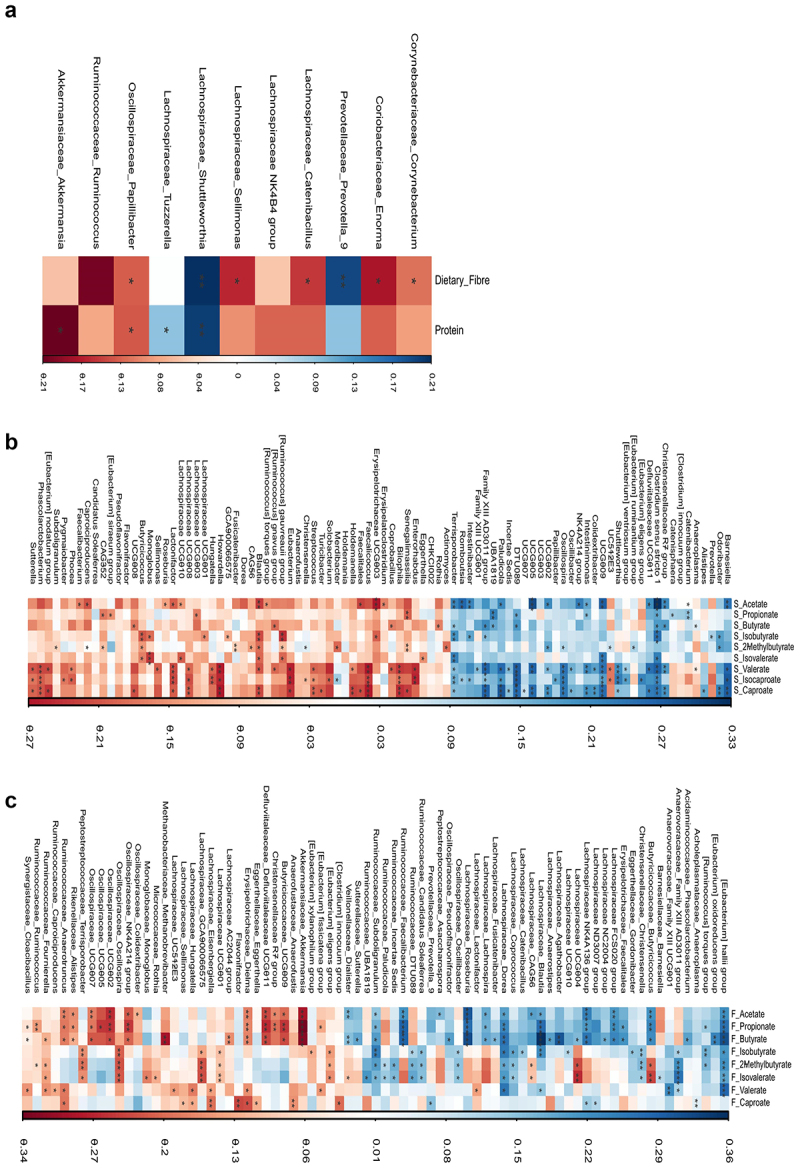
Spearman correlations among bacterial genera, SCFAs, and their precursors dietary fibers (DF) and proteins. (a) Bacterial genera significantly correlating with DF and protein intake. (b) Bacterial genera correlations with serum SCFAs (c) Bacterial genera correlations with fecal SCFAs. Significant correlations are presented as *=p < 0.05, **=p < 0.01 and ***=p < 0.001.

DF intake was negatively correlated with the presence of several bacterial genera (*p* < 0.05), including *Corynebacterium*, *Enorma*, *Catenibacillus*, *Sellimonas*, and *Papillibacter* (Figure 6(a)). However, *Prevotella* and *Shuttleworthia* abundance increased (*p* < 0.05) with an increase in DF intake (Figure 6(a)). Significant correlations were also found between several bacterial genera and circulating (Figure 6(b)), and fecal SCFA levels (Figure 6(c)). Correlations in serum were more significant (*p* < 0.01) for C5-C6 SCFAs; however, in feces, C2-C4 (acetate, propionate, and butyrate) showed stronger correlations (*p* < 0.01) than other SCFAs.

Major SCFA-producing bacteria including *Butyricicoccus*, *Dorea*, *Blautia*, and *Sutterella* were positively (*p* < 0.05) correlated with SCFAs (C2-C4) in the feces but negatively correlated with them in the serum (*p* > 0.05); except for *Butyricicoccus* which showed a consistent negative correlation (*p* < 0.05) with branched SCFAs (C4, C5) in both serum and feces (Figure 6(b,c)).

### 3.5. Role of *APOE4* genotype and dietary fibre intake in altering gut-brain and gut-metabolic modules

**Figure 7.**
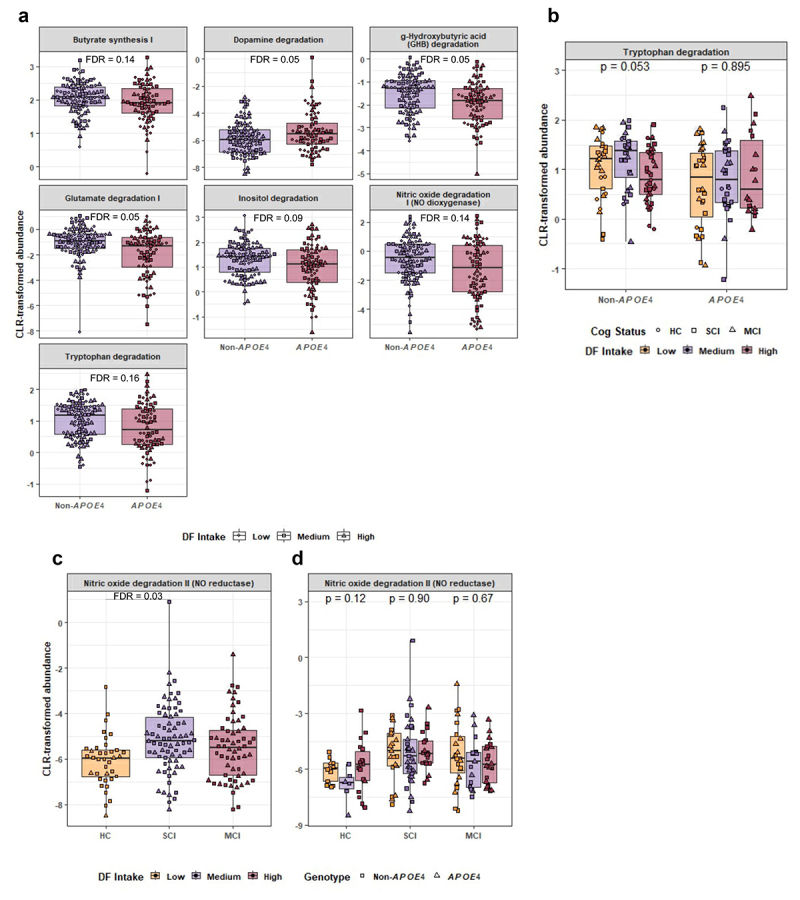
Differentially abundant gut-brain modules (GBMs) and their association with dietary fiber (DF) intake. (a) Differentially abundant gut-brain metabolic pathways between non-*APOE4* and *APOE4* carriers. Linear regression analyses were performed on centered log ratio (CLR) transformed data. *p* values are reported following Benjamini & Hochberg FDR correction. (b) Tryptophan degradation in non-*APOE4* and *APOE4* carriers based on DF intake. Kruskal–Wallis tests were used to compare means based on DF intake within each genotype and *p* values are mentioned on the plot. (c) Nitric oxide degradation in SCI (subjective cognitive impairment) and MCI (mild cognitive impairment) participants vs HC (healthy controls). Linear regression analysis was performed, and *p* values are reported following Benjamini & Hochberg FDR correction. (d) Nitric oxide degradation in SCI and MCI participants vs HC based on DF intake. Kruskal–Wallis tests were used to compare means based on DF intake within each cognitive group. Non-*APOE4* and *APOE4* carriers are presented with different shapes in both plots.

*APOE4* genotype was associated with significantly altered gut-brain modules (Figure 7(a)). *APOE4* carriers had significantly lower representation of butyrate synthesis I (FDR = 0.14), and degradation of gamma-hydroxybutyric acid (GHB) (FDR = 0.05), glutamate I (FDR = 0.05), inositol (FDR = 0.09), nitric oxide I (FDR = 0.14), and tryptophan (FDR = 0.13) as compared to non-*APOE4* carriers (Figure 7(a)). *APOE4* carriers showed a higher predicted degradation of dopamine as compared to non-*APOE4* carriers (FDR = 0.05). These pathways were confirmed by significant KO enzyme differences in both *APOE4* and non-*APOE4* carriers (Supp Fig S3).

DF intake did not show any significant role in altering these pathways within either genotype, but a trend toward lower tryptophan degradation (*p* = 0.05) was observed with high DF intake in non-*APOE4* carriers only (Figure 7(b)). The cognitive status of the participants was significantly associated with nitric oxide (NO) degradation via NO reductase; and SCI participants reported the highest comparative degradation (Figure 7(c)), however this was not affected by DF intake (Figure 7(d)).

Several Gut-Metabolic modules (GMMs) were also significantly influenced by *APOE4* genotype (Supp Fig S4) and DF intake (Supp Fig S5).

### 3.6. *APOE4* carriers show negative association between serum SCFAs and their predicted synthesis in the gut

Functional predictions from the PICRUSt2 output showed lower levels of butyrate synthesis in *APOE4* carriers (Figure 7(a)), confirmed by significantly lower predicted levels of essential enzymes involved in butyrate synthesis, i.e. butyryl CoA dehydrogenase, phosphate butyryl transferase, and butyrate kinase (Supp Fig S3). *APOE4* carriers showed a negative association between serum acetate, propionate, and butyrate, and the predicted capacity of gut microbiota to produce these SCFAs (Supp Fig S6). In *APOE4* carriers, serum acetate concentration was negatively (*p* < 0.05) associated with the GBM acetate synthesis I (representing acetate synthesis from acetyl-CoA I), whereas non-*APOE4* carriers showed a slightly positive association (Supp Fig S6).

*APOE4* carriers also showed similar negative association for serum propionate (*p* = 0.05) and butyrate (*p* = 0.04) with propionate synthesis II (synthesis from acetate and lactate via propanoyl-CoA) and butyrate synthesis II (synthesis from acetate and butanoate via crotonyl-CoA and Acetyl-CoA), respectively, whereas non-*APOE4* carriers showed positive associations (Supp Fig S6). Linear regression analysis was performed to explore these relationships, and no significant associations were observed between fecal levels of SCFAs and their predicted synthesis by gut bacteria.

## 4. Discussion

*APOE* genotype influences various physiological processes,^40,41^ including gut microbiota composition^6,7^ and microbe-associated-metabolites like SCFAs.^6,42^ However, its interaction with dietary intake and metabolism, particularly DF (a primary substrate for gut microbiota), remains unclear. Our study is the first to suggest that DF intervention as a microbiota-targeted approach may help prevent cognitive impairment, but only in non-*APOE4* carriers.

*APOE4* carriers had lower DF intake, possibly due to *APOE’s* role in metabolism,^43^ which may influence dietary preferences resulting in *APOE4* carriers having an aversion for certain foods including those rich in DF. Alternatively, a differential gut microbiota could drive these dietary behavior changes.^44^ We observed differential gamma-hydroxybutyric acid (GHB) degradation between *APOE4* and non-*APOE4* carriers, which is known to influence dietary behaviors.^45,46^ Further research is needed to understand the mechanisms behind reduced DF intake in *APOE4* carriers.

It is important to establish if the differences in DF consumption also drive variations in DF-derived metabolites like SCFAs. Previous research on DF intake and SCFA levels has been inconsistent, with some studies finding correlations, while others do not. Vinelli and colleagues conducted a systematic review of 44 studies and found that 26 of these studies reported no significant differences in individual SCFA levels.^47^ SCFAs profile depends on the dose,^48,49^ and type and structure of DF consumed,^47,49,50^ and a lack of fermentable fibers may shift fermentation toward protein, producing branched-chain fatty acids.^51^ Other bioactives, like polyphenols also influence SCFAs production by shaping gut microbiota.^52^ We focused mainly on DFs as primary precursors for SCFAs, and our analysis suggests that the differences in serum butyrate, valerate, and caproate between *APOE4* and non-*APOE4* carriers are independent of DF intake, aligning with our previous findings on genotype-associated SCFAs variations.^6^

Previous studies, including our own, have linked gut microbial diversity to the *APOE* genotype.^3,5,6,42^ Our findings further reveal distinct gut bacterial diversity between *APOE4* and non-*APOE4* carriers, potentially explaining the lower SCFA-producing bacteria and serum SCFA levels in *APOE4* carriers. The predicted SCFA-producing capacity of the gut microbiota was significantly associated with serum acetate, propionate, and butyrate levels, but this relationship differed by *APOE4* genotype. In non-*APOE4* carriers, microbial SCFA synthesis aligned with serum SCFA levels, whereas *APOE4* carriers showed an opposite trend (Supp Fig S6). This suggests metabolic differences between *APOE4* and non-*APOE4* carriers, warranting further investigation into the underlying mechanisms.

Strong evidence supports the role of DF in shaping gut microbiota,^16,49^ yet its interaction with *APOE4* genotype remains unclear. Our findings show that DF intake significantly correlates with gut microbial diversity only in non-*APOE4* carriers, suggesting that metabolic disruptions linked to *APOE4* genotype^53,54^ may influence DF metabolism. Further analysis highlights significant bacterial differences in response to DF intake exclusively in non-*APOE4* carriers, reinforcing the idea that *APOE4* carriers are relatively unresponsive to DF-driven gut microbiota changes.

Higher DF intake in non-*APOE4* carriers is associated with decrease in *Corynebacterium*, a bacteria linked to conditions such as depression,^55^ schizophrenia,^56^ and autism spectrum disorders.^57^ DF intake also correlated with *Defluviitaleaceae UCG-011* in non-*APOE4* carriers, which may help maintain cognitive function through the gut-brain axis.^58^ Additionally, *Bilophila*, *Eubacterium eligens*, *Sutterella*, *Bacteroides*, and *Parabacteroides* were associated with DF intake in non-*APOE4* carriers. *Eubacterium eligens group* has been linked to improved cognitive function and increased SCFA production,^59^ while *Parabacteroides*, known for acetate production, helps prevent inflammation.^60^ The changes in bacterial abundances were not always linear, potentially due to differences in DF composition, which we could not account for due to limited data availability, as both cohorts used different FFQs which used AOAC fiber and NSP fiber for DF quantification in CANN and COMBAT cohorts, respectively.

DF-derived bacterial changes in non-*APOE4* carriers predict better cognitive health. We found that non-*APOE4* carriers are more responsive to changes in DF intake and subsequently SCFA levels in SCI and MCI compared to HC. Further research is needed to determine whether these differences stem from distinct microbiota between *APOE4* and non-*APOE4* carriers or unique metabolic mechanisms that shape microbiota composition. DF-derived SCFAs modulate the intestinal permeability^61^ which could be another potential contributor to gut microbiota linked cognitive decline in *APOE4* carriers. DF-derived SCFAs support microbial cross-feeding, influencing overall gut metabolism.^62^ Nitric oxide (NO), a key microbial metabolite, is involved in neuronal signaling,^63^ synaptic plasticity, memory function,^64^ and cerebral blood flow regulation.^65^ Elevated NO degradation in SCI group may contribute to cognitive decline, potentially preventable by enhancing NO production. Gut microbiota can mediate NO production via dissimilatory nitrate reduction to ammonia (DNRA) pathway,^66^ and we observed a significant increase in dissimilatory nitrate reduction with higher DF intake (Supp Fig. S5a). While this was not associated with cognitive status or *APOE* genotype (Supp Fig. S5b), we propose that high DF intake might support NO production, potentially counteracting NO degradation in SCI through SCFA-driven cross-feeding.

Moreover, reduced straight SCFAs have been reported in the feces of MCI individuals vs healthy controls and have been suggested as early diagnostic biomarkers for MCI and potential targets for disease prevention.^67^ Most of the other studies also report lower fecal SCFA levels in neurocognitive disorders like anorexia nervosa, Parkinson’s disease (PD), AD, and depression.^68^ However, fecal concentrations of SCFAs are neither a true representative of SCFA production nor circulating levels, as confirmed by Chen et al., in patients with PD who presented lower fecal but higher plasma concentrations of acetate.^69^ This low concordance between fecal and serum SCFAs has also been reported in other cohorts (TwinsUK and ZOE PREDICT).^70^ Moreover, we observed that individuals with differing cognitive statuses exhibited more pronounced differences in serum SCFA concentrations compared to fecal SCFA levels. Although most of the literature reports fecal levels of SCFAs, we did not find any correlation between fecal SCFA and DF intake. Interestingly, serum C5 and C6 SCFAs showed significant positive correlation with DF intake, proposing that these SCFAs may not be entirely metabolized in the gut or the liver. Hence, their elevated serum levels potentially reflecting higher DF consumption, higher production in the gut and ultimately faster absorption in the circulation. The majority of SCFAs produced in the gut are utilized locally as an energy substrate by gut epithelial cells with the remainder absorbed into the portal circulation, where most of the C2-C4 SCFAs are metabolised in the liver.^12^ These results align with previous findings reported by Cummings and colleagues, which showed that the levels of acetate, propionate, and butyrate are approximately five times lower in peripheral venous blood (79 µmol/l) compared to portal blood (375 µmol/l),^71^ indicating significant utilization of these SCFAs in the liver. However, it proposes that SCFAs (C5-C6) tend to enter the systemic circulation (as indicated by the positive correlation between serum and fecal levels), where they are eventually metabolized into smaller SCFAs (C2-C4) which have the most physiological effects.

Among all SCFAs, butyrate has the greatest impact at physiological level, thus posing higher relevance for butyrate producing bacteria.^72^ *Lachnospiraceae* and *Ruminococcaceae* families have bacteria mainly known for butyrate production,^72^ but also produce other SCFAs like other genera including *Roseburia*,^73^ *Blautia*,^74^ *E.hallii*,^75,76^ and *Faecalibacterium*.^77^ We observed these genera to be positively correlated with fecal SCFAs, but contrarily, *Roseburia, Blautia*, and *Faecalibacterium* were negatively correlated with the serum levels of SCFAs. These conflicting associations with serum and fecal SCFA levels have also been reported by other studies,^69,78^ supporting that fecal SCFAs are not a true representation of physiologically circulating levels highlighting the need to focus more on systemic concentrations.

Complex gut-bacterial cross-feeding mechanisms may drive DF-induced microbiome shifts in non-*APOE4* carriers, potentially altering gut metabolism. For instance, we predicted significantly lower tryptophan degradation in *APOE4* carriers, which was further confirmed by a lower predicted abundance of tryptophanase enzyme in *APOE4* carriers (Supp Fig S3). Tryptophan degradation is associated with the synthesis of serotonin and other metabolites involved in the modulation of neurotransmission and elimination of amyloid-β peptides from the brain.^69,78^ Previous research has reported changes in tryptophan metabolism in non-*APOE4* mice in response to inulin supplementation, while no such alterations were noted in *APOE4* mice.^4^ It may therefore be speculated that the effect of DF on altered tryptophan metabolism may be independent of tryptophan content and rather dependent on DF-derived microbial cross-feeding of microbes involved in tryptophan metabolism,^79^ which are also shaped by the *APOE* genotype.

Our novel findings indicate that non-*APOE4* carriers tend to consume more DFs and are more responsive to DF-related microbial changes than *APOE4* carriers. DF-derived metabolites (i.e. SCFAs) and their associations with cognitive status were also found to be more strongly associated in non-*APOE4* carriers. Non-*APOE4* carriers show positive association with predicted SCFAs synthesis and their serum levels, as opposed to negative associations in *APOE4* carriers. These findings suggest that *APOE4* and non-*APOE4* carriers may exhibit distinct physiological responses, potentially reflecting divergent metabolic pathways. Overall, we suggest that DF-induced changes in the microbiome and resulting serum SCFAs may be an effective strategy for preventing cognitive decline, but primarily in non-*APOE4* carriers. In an era of move toward a more personalized approach to disease prevention and therapeutics, our results underscore the potential for the targeting of increased fiber intake recommendations to support long-term cognitive health.

However, some limitations should be stressed along with potential areas of future research endeavors. This is a single center study which may not represent the diversity of different populations and dietary pattens. In addition, a larger sample size would have greatly strengthened the validity of our results; however, the level of significance and physiological trends observed offer a compelling reason for further research to consolidate these findings in a larger population. Also, our analysis includes total DF; it did not consider the individual types of dietary fiber, which may differentially affect the gut microbiota composition and subsequent metabolites. Although all the fecal samples were stored and processed using standardized protocols, variability introduced by at-home collection and transport to the research facility may have impacted the levels of SCFAs, given their volatile nature. Additionally, although the groups were matched for sex, age and BMI unaccounted for potentially confounding factors such as comorbid health conditions, medication use, and levels of physical activity may have influenced the observed outcomes and associations, which should be considered in future research. Finally, controlled DF intervention studies could further clarify the causal or associative link among the *APOE4* genotype, gut microbiota, DF intake, related metabolites, and cognitive benefits.

## Supplementary Material

Liaquat et al_CANN_Supplementary rev1.docx

## Data Availability

The 16S rRNA gene sequence data have been deposited in the NCBI BioProject database (https://www.ncbi.nlm.nih.gov/bioproject/.) under accession number PRJNA1109848 (https://dataview.ncbi.nlm.nih.gov/object/PRJNA1109848?reviewer=6ap0u8gbeurjsalfs5141rrcvn). Other data that support the findings of this study are available from the corresponding authors upon reasonable request.
