# Supplementary material for: APOE4 genotype shapes the role of dietary fibers in cognitive health through gut microbiota changes
Source: Gut Microbes. 2025 Jul 2;17(1):2526133. doi: 10.1080/19490976.2025.2526133 (PMC12233883; doi:10.1080/19490976.2025.2526133)
Supplement: Liaquat et al_CANN_Supplementary rev1.docx [file KGMI_A_2526133_SM8044.docx]

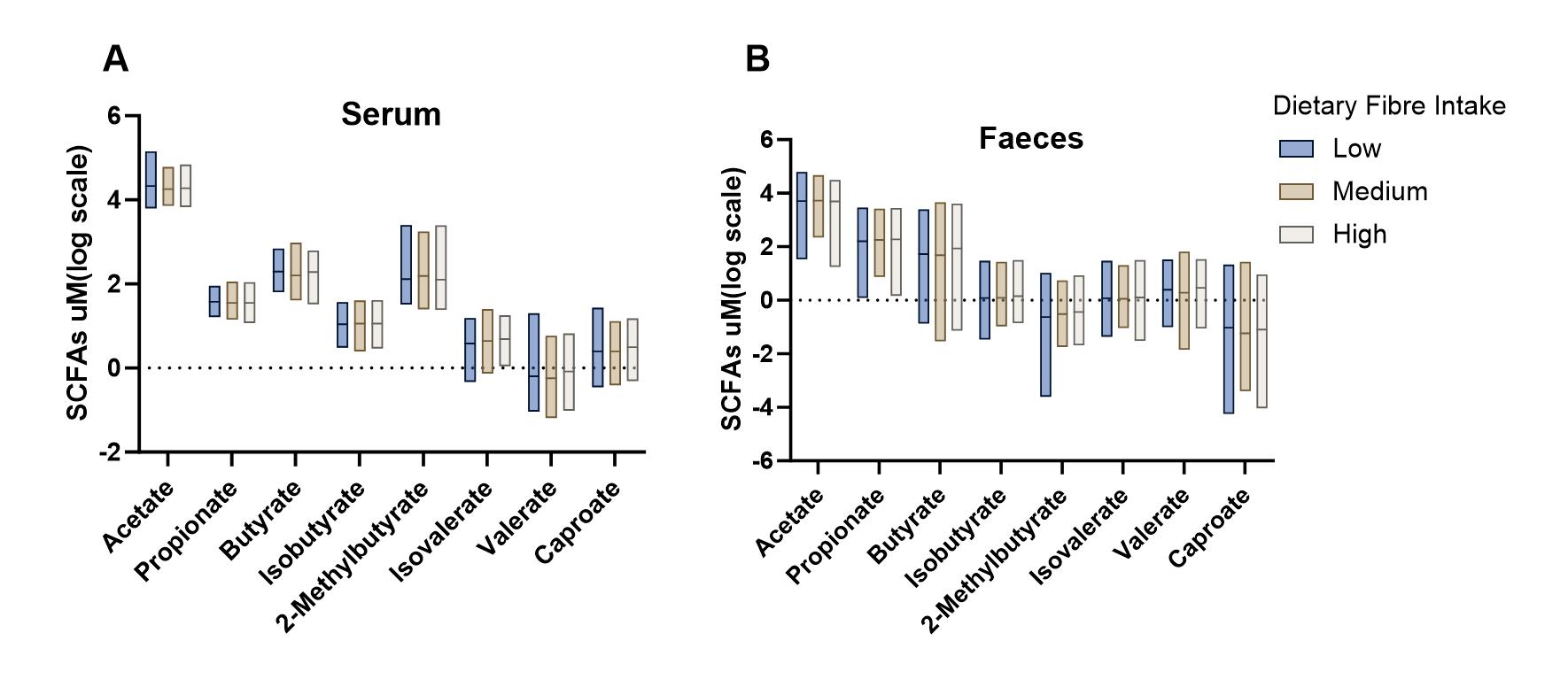

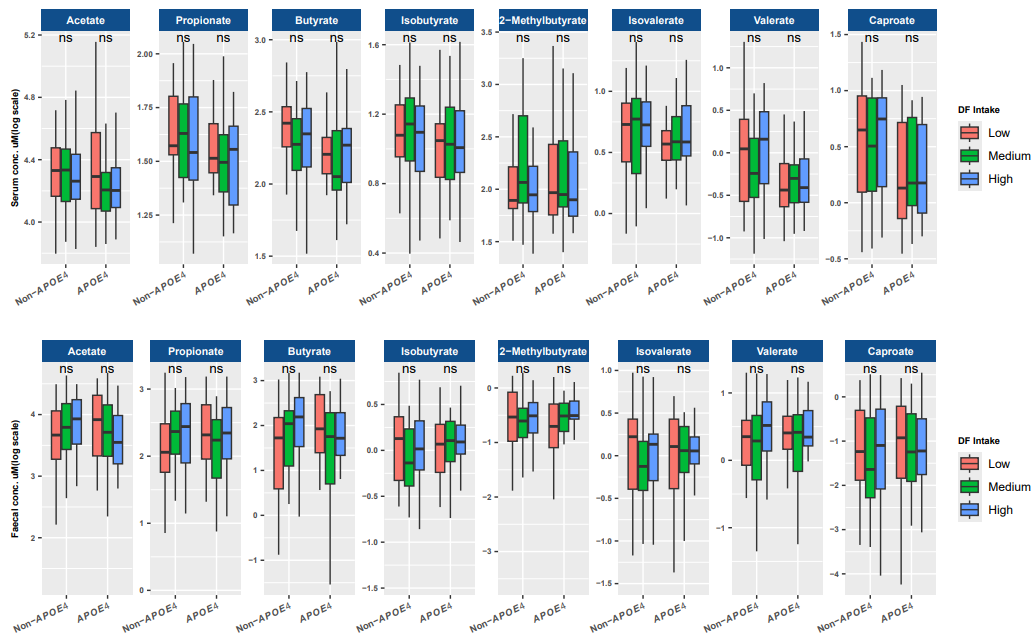


**D**

**C**

Supp Fig 1| **Association between dietary fibre (DF) intake, *APOE4* genotype, and SCFAs concentrations. A)** Serum and **B)** Faecal SCFAs concentrations *(µ*M log scaled) in participants with low, medium, and high DF consumption. **C)** Serum SCFAs concentrations (*µ*M log scaled) in people with non-*APOE4* and *APOE4* genotype and split based on their DF intake. **D)** Faecal SCFAs concentrations (*µ*M log scaled) in people with non-*APOE4* and *APOE4* genotype and split based on their DF intake. Kruskal-Wallis test was performed for comparisons and significance levels are presented on the plots; ns = non-significant (p>0.05).


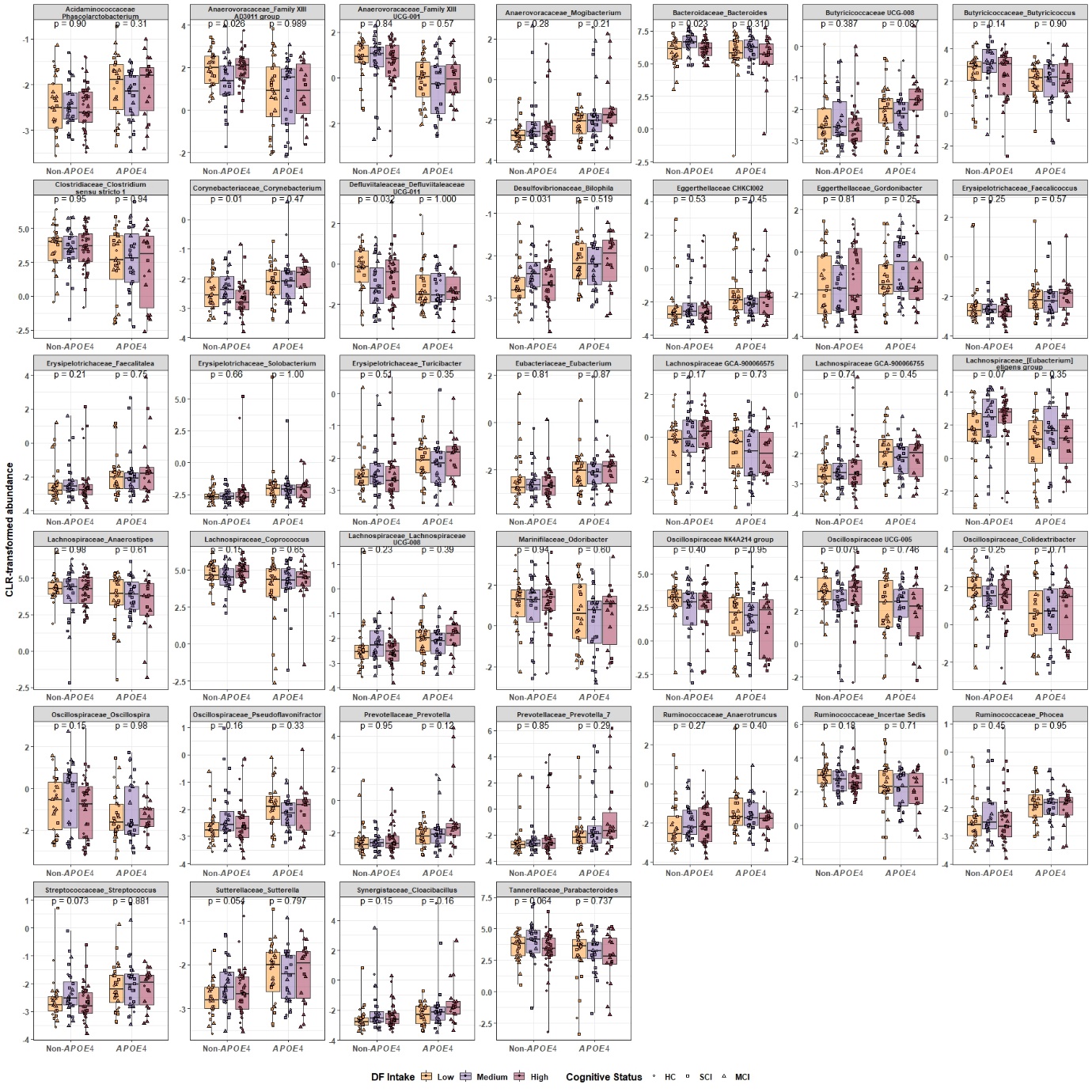


Supp Fig 2 | **Differentially abundant bacterial genera within non-*APOE4* and *APOE4* carriers split by dietary fibre (DF) intake.** Kruskal-Wallis test was performed to compare the effect of DF intake within each genotype, p values are reported on individual boxplots.

Supp Fig 3| **PICRUSt2 predicted KEGG Orthologue (KO) enzymes abundance in non-*APOE4* and *APOE4* carriers.** Data are presented as mean ±SEM of enzyme abundance values (log 10 transformed). Multiple Mann-Whitney tests were used to compare groups followed by Benjamini and Hochberg FDR correction. * = p<0.05.


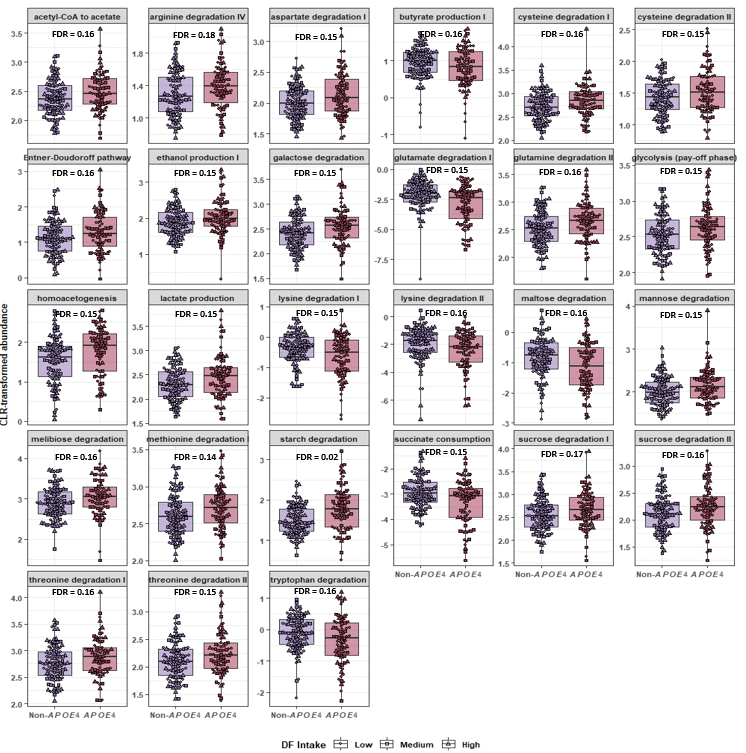
Supp Fig 4| **Differentially abundant Gut-Metabolic Modules (GMMs) based on *APOE4* genotype and the association with dietary fibre (DF) intake**. Linear regression analysis was performed, and p values are reported following Benjamini & Hochberg FDR correction.


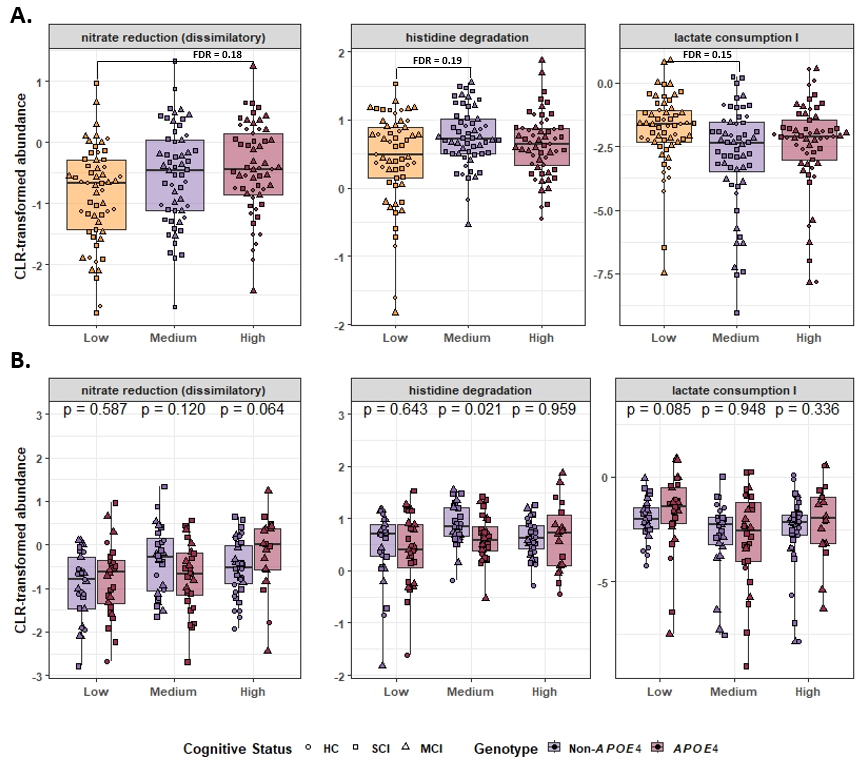
Supp Fig 5| **Differentially abundant Gut-Metabolic Modules (GMMs) based on dietary fibre (DF) intake and the association with *APOE4* genotype**. **A)** Differentially abundant Gut-Metabolic modules based on DF intake. Linear regression analysis was performed, and p values are reported following Benjamini & Hochberg FDR correction. **B)** Role of *APOE4* genotype within each group. Kruskal-Wallis tests were used to compare means based on genotype within each DF intake group and p values are mentioned on the plots.


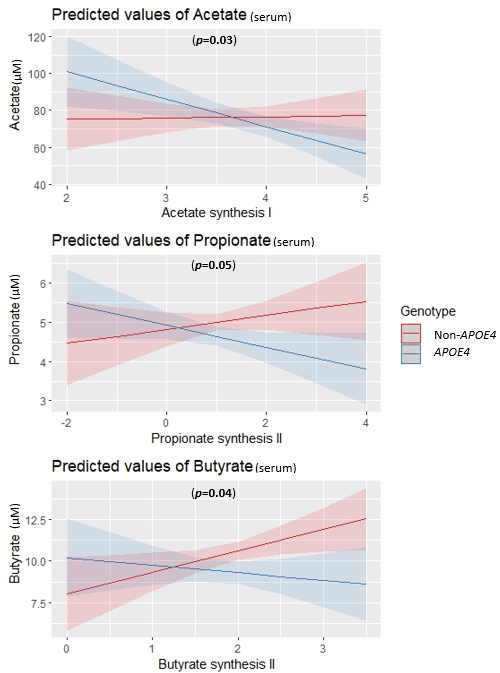
Supp Fig 6| **Association between Gut-Brain Modules (predicting SCFAs syntheses) and serum SCFA levels.** Linear regression analysis was performed between predicted SCFAs synthesis (x-axis) and their serum levels (y-axis), and significant interactions (*p*<0.05) between GBM modules for SCFAs synthesis and *APOE4* genotype are plotted for respective SCFAs.

Supp Table 1| **Differentially abundant bacterial genera within non-*APOE4* and *APOE4* carriers**

| Genus | Genotype | Mean | SD | | FDR * |
| --- | --- | --- | --- | --- | --- |
| *Phascolarctobacterium* | *APOE4* | -2.138 | 0.629 | 0.03 | |
|  | Non-*APOE4* | -2.466 | 0.528 |  | |
| *Family XIII AD3011 group* | *APOE4* | 0.795 | 1.578 | 0.00 | |
|  | Non-*APOE4* | 1.770 | 0.995 |  | |
| *Family* *XIII* *UCG-001* | *APOE4* | -0.232 | 1.177 | 0.00 | |
|  | Non-*APOE4* | 0.692 | 1.087 |  | |
| *Mogibacterium* | *APOE4* | -1.753 | 1.350 | 0.02 | |
|  | Non-*APOE4* | -2.399 | 1.116 |  | |
| *Bacteroides* | *APOE4* | 5.782 | 1.554 | 0.04 | |
|  | Non-*APOE4* | 6.176 | 0.885 |  | |
| *Butyricicoccus* | *APOE4* | 2.043 | 1.243 | 0.08 | |
|  | Non-*APOE4* | 2.528 | 1.489 |  | |
| *Butyricicoccaceae_UCG-008* | *APOE4* | -2.004 | 0.781 | 0.02 | |
|  | Non-*APOE4* | -2.444 | 0.794 |  | |
| *Clostridium sensu stricto 1* | *APOE4* | 2.426 | 2.554 | 0.03 | |
|  | Non-*APOE4* | 3.531 | 1.567 |  | |
| *Corynebacterium* | *APOE4* | -2.088 | 0.702 | 0.02 | |
|  | Non-*APOE4* | -2.504 | 0.620 |  | |
| *Defluviitaleaceae UCG-011* | *APOE4* | -1.198 | 1.050 | 0.06 | |
|  | Non-*APOE4* | -0.631 | 1.267 |  | |
| *Bilophila* | *APOE4* | -2.184 | 0.592 | 0.00 | |
|  | Non-*APOE4* | -2.624 | 0.519 |  | |
| *Eggerthellaceae_CHKCI002* | *APOE4* | -1.662 | 1.529 | 0.08 | |
|  | Non-*APOE4* | -2.187 | 1.449 |  | |
| *Gordonibacter* | *APOE4* | -1.082 | 1.429 | 0.10 | |
|  | Non-*APOE4* | -1.542 | 1.485 |  | |
| *Faecalicoccus* | *APOE4* | -2.084 | 0.959 | 0.08 | |
|  | Non-*APOE4* | -2.560 | 0.933 |  | |
| *Faecalitalea* | *APOE4* | -1.779 | 1.486 | 0.10 | |
|  | Non-*APOE4* | -2.361 | 1.201 |  | |
| *Solobacterium* | *APOE4* | -1.933 | 1.386 | 0.06 | |
|  | Non-*APOE4* | -2.485 | 1.123 |  | |
| *Turicibacter* | *APOE4* | -2.121 | 0.696 | 0.06 | |
|  | Non-*APOE4* | -2.456 | 0.792 |  | |
| *Eubacterium* | *APOE4* | -2.028 | 0.920 | 0.04 | |
|  | Non-*APOE4* | -2.486 | 0.840 |  | |
| *[Eubacterium] eligens group* | *APOE4* | 1.132 | 1.868 | 0.04 | |
|  | Non-*APOE4* | 2.012 | 1.753 |  | |
| *Anaerostipes* | *APOE4* | 3.558 | 1.638 | 0.10 | |
|  | Non-*APOE4* | 4.123 | 1.260 |  | |
| *Coprococcus* | *APOE4* | 3.994 | 1.791 | 0.03 | |
|  | Non-*APOE4* | 4.705 | 0.757 |  | |
| *Lachnospiraceae_GCA-900066575* | *APOE4* | -0.680 | 1.229 | 0.08 | |
|  | Non-*APOE4* | -0.155 | 1.316 |  | |
| *Lachnospiraceae UCG-008* | *APOE4* | -2.066 | 0.676 | 0.10 | |
|  | Non-*APOE4* | -2.375 | 0.750 |  | |
| *Odoribacter* | *APOE4* | 0.452 | 1.622 | 0.05 | |
|  | Non-*APOE4* | 1.086 | 1.262 |  | |
| *Colidextribacter* | *APOE4* | 0.652 | 1.674 | 0.02 | |
|  | Non-*APOE4* | 1.527 | 1.231 |  | |
| *Oscillospiraceae_NK4A214 group* | *APOE4* | 1.570 | 2.206 | 0.03 | |
|  | Non-*APOE4* | 2.613 | 1.918 |  | |
| *Oscillospira* | *APOE4* | -1.295 | 1.188 | 0.08 | |
|  | Non-*APOE4* | -0.729 | 1.467 |  | |
| *Pseudoflavonifractor* | *APOE4* | -2.032 | 0.762 | 0.03 | |
|  | Non-*APOE4* | -2.470 | 0.856 |  | |
| *Oscillospiraceae_UCG-005* | *APOE4* | 2.179 | 1.637 | 0.10 | |
|  | Non-*APOE4* | 2.839 | 1.409 |  | |
| *Prevotella* | *APOE4* | -1.823 | 1.441 | 0.02 | |
|  | Non-*APOE4* | -2.446 | 0.827 |  | |
| *Prevotella_7* | *APOE4* | -1.301 | 2.232 | 0.03 | |
|  | Non-*APOE4* | -2.192 | 1.689 |  | |
| *Anaerotruncus* | *APOE4* | -1.577 | 0.960 | 0.06 | |
|  | Non-*APOE4* | -2.029 | 1.059 |  | |
| *Incertae Sedis* | *APOE4* | 2.160 | 1.387 | 0.02 | |
|  | Non-*APOE4* | 2.807 | 0.826 |  | |
| *Phocea* | *APOE4* | -1.930 | 0.649 | 0.03 | |
|  | Non-*APOE4* | -2.370 | 0.899 |  | |
| *Streptococcus* | *APOE4* | -2.091 | 0.848 | 0.03 | |
|  | Non-*APOE4* | -2.545 | 0.727 |  | |
| *Sutterella* | *APOE4* | -2.200 | 0.635 | 0.01 | |
|  | Non-*APOE4* | -2.599 | 0.551 |  | |
| *Cloacibacillus* | *APOE4* | -1.924 | 1.348 | 0.08 | |
|  | Non-*APOE4* | -2.377 | 1.023 |  | |
| *Parabacteroides* | *APOE4* | 2.966 | 1.860 | 0.03 | |
|  | Non-*APOE4* | 3.592 | 1.459 |  | |

Data is reported as mean and SD of the centered log ratio (CLR) transformed abundance of each bacterial genera. Linear regression model was used for each genera and *p* values for all the contrasts were corrected for false discovery rate (FDR) using Benjamini & Hochberg’s procedure. q-value of 0.1 was used as a cut-off for statistical significance.

Supp Table 2| **Age, Sex and BMI comparisons between non-*APOE4* and *APOE4* carriers in DF consumer groups**

|  | Overall | Low DF Consumers  (n=57) | | | Medium DF Consumers  (n=56) | | | High DF Consumers  (n=57) | | |
| --- | --- | --- | --- | --- | --- | --- | --- | --- | --- | --- |
|  |  | **Non-*APOE4* carriers** | ***APOE4* carriers** | ***P*- values** | **Non-*APOE4* carriers** | ***APOE4* carriers** | ***P*-values** | **Non-*APOE4* carriers** | ***APOE4* carriers** | ***P*-values** |
| Age (y) | 65.8  (6.0) | 65.2  (6.7) | 64.8  (6.3) | 0.73 | 66.4  (4.4) | 66.0  (6.4) | 0.73 | 65.8  (6.1) | 66.6  (6.0) | 0.57 |
| Sex, M/F (%F) | 75/95 (56%) | 7/22  (76%) | 12/16  (57%) | 0.03 | 15/13  (46%) | 11/17  (61%) | 0.08 | 20/19  (49%) | 10/8  (44%) | 0.67 |
| BMI (kg/m^2^) | 25.7  (3.7) | 24.9  (3.0) | 28.0  (4.5) | 0.20 | 25.1  (3.0) | 25.9  (4.6) | 0.29 | 25.4  (2.8) | 25.2  (3.7) | 0.85 |

Data are mean (SD) or as stated otherwise.

DF, dietary fibre; T-test was used to compare between-genotype differences for Age and BMI within each group, and Chi-square test was used for sex comparisons.
